# A look‐locker acquisition scheme for quantitative myocardial perfusion imaging with FAIR arterial spin labeling in humans at 3 tesla

**DOI:** 10.1002/mrm.26388

**Published:** 2016-09-08

**Authors:** Graeme A. Keith, Christopher T. Rodgers, Michael A. Chappell, Matthew D. Robson

**Affiliations:** ^1^ Oxford Centre for Clinical Magnetic Resonance Research University of Oxford, John Radcliffe Hospital Oxford United Kingdom; ^2^ Institute of Biomedical Engineering University of Oxford, Old Road Campus Oxford United Kingdom

**Keywords:** ASL, MBF, FAIR, heart, human

## Abstract

**Purpose:**

A novel method for quantitative measurement of myocardial blood flow (MBF) using arterial spin labeling (ASL) in a single breath‐hold is presented, evaluated by simulations, phantom studies and in vivo studies and tested for reproducibility and variability.

**Methods:**

A flow‐sensitive alternating inversion recovery (FAIR) ASL method with Look‐Locker readout (LL‐FAIR‐ASL) was implemented at 3 tesla. Scans were performed on 10 healthy volunteers and MBF measured in three slices. The method was investigated for reproducibility by Bland‐Altman analysis and statistical measures, the coefficients of reproducibility (CR) and variation (CV) are reported.

**Results:**

The MBF values for the basal, mid, and apical slices were 1.04 ± 0.40, 1.06 ± 0.46, and 1.06 ± 0.38 ml/g/min, respectively (mean ± SD), which compare well with literature values. The CV across all scans, 43%, was greater than the between‐session and within‐session values, at 16 and 13%, respectively, for the mid‐ventricular slice. The change in MBF required for detection, from the CR, was 61% between‐session and 53% within‐session for the mid‐ventricle.

**Conclusion:**

This study shows the feasibility of the LL‐FAIR‐ASL method for the quantification of MBF. The statistical measures reported will allow the planning of future clinical research studies involving rest and stress measurements. Magn Reson Med 78:541–549, 2017. © 2016 The Authors Magnetic Resonance in Medicine published by Wiley Periodicals, Inc. on behalf of International Society for Magnetic Resonance in Medicine. This is an open access article under the terms of the Creative Commons Attribution License, which permits use, distribution and reproduction in any medium, provided the original work is properly cited.

## INTRODUCTION

Cardiovascular magnetic resonance (CMR) imaging is a powerful tool for the investigation of common pathologies of the heart such as congenital heart disease, coronary artery disease, and cardiomyopathies 1. One of the most useful processes that CMR allows us to investigate is the perfusion of blood in the myocardial tissue. In clinical practice, this is achieved with the use of an intravenous extracellular contrast agent, such as Gadolinium‐DPTA 2, where the transit of the agent through the capillary bed of the myocardium can be observed. These first‐pass techniques provide clinically applicable methods of imaging perfusion, but suffer from several drawbacks, such as significant problems with image artifacts 3, cost, the risk of the contrast agent itself to patients with renal conditions 4, and the difficulty of performing multiple serial evaluations owing to the lingering presence of the contrast agent.

An attractive alternative to first‐pass perfusion CMR techniques and other, nuclear based modalities, such as single‐photon emission computed tomography (SPECT) 5, 6 or positron emission tomography (PET) 7, 8, would be to use arterial spin labeling (ASL). ASL is a noninvasive technique which uses magnetic labeling strategies to allow the proton spins present in the blood water to act as an endogenous contrast agent 9, 10. In one of the simplest ASL experiments, known as flow‐sensitive alternating inversion recovery (FAIR) 11, 12, two images or sets of images are collected, one in which a globally selective (GS) inversion pulse is applied before the readout (the control image) such that the spins of the myocardial tissue of interest and those of the inflowing blood are both inverted. The second image set is preceded by a slice‐selective (SS) inversion through a slice of the myocardium (the tag or label image) such that the myocardial tissue in the imaging slice is again inverted but the blood water spins flowing into the image slice remain in thermomagnetic equilibrium. The effect of the presence of these inflowing spins in the SS experiment is to cause an apparent shortening of the longitudinal relaxation time (*T*_1_) of the tissue. It is the size of the difference in the observed *T*_1_ values between the two measurements that allows us to quantitatively evaluate the blood flow to the myocardium.

The measurement of *T*_1_ values in the myocardium, often in the form of *T*_1_ mapping techniques, has emerged as a useful tool in CMR 13. One established, robust method often used for *T*_1_ mapping is the modified Look‐Locker inversion recovery (MOLLI) 14, 15, 16 sequence and it's variants, which allow for the measurement and parametric mapping of the myocardial *T*_1_ in vivo, in a single breath‐hold. In this approach, an inversion pulse is applied and the relaxation of the longitudinal magnetization is sampled following an inversion time, TI, then again following the equivalent of one R–R interval, measured by electrocardiograph (ECG) ‐gating, and so on to build up a relaxation curve allowing the quantification of *T*_1_. Different variants of the technique use different inversion‐readout schemes (3(3)3(3)5 for MOLLI, 5(1)1(1)1 for ShMOLLI and so on, indicating the number of look‐locker images acquired and the duration of the gaps between acquisitions, all in units of heart beats).

In this work, a method for measuring myocardial blood flow (MBF) noninvasively using a FAIR labeling scheme combined with a Look‐Locker acquisition (LL‐FAIR‐ASL) 17 is presented. The described method allows for the acquisition of both FAIR inversion states (SS and GS) within a single breath‐hold. The method is assessed by way of simulation, phantom study, and in vivo application. The method is further used for quantitative analysis of MBF in healthy volunteers. MBF is measured in three slices in humans with ASL for the first time and is assessed for reproducibility and variability.

## METHODS

### Sequence Design

**Figure 1:**
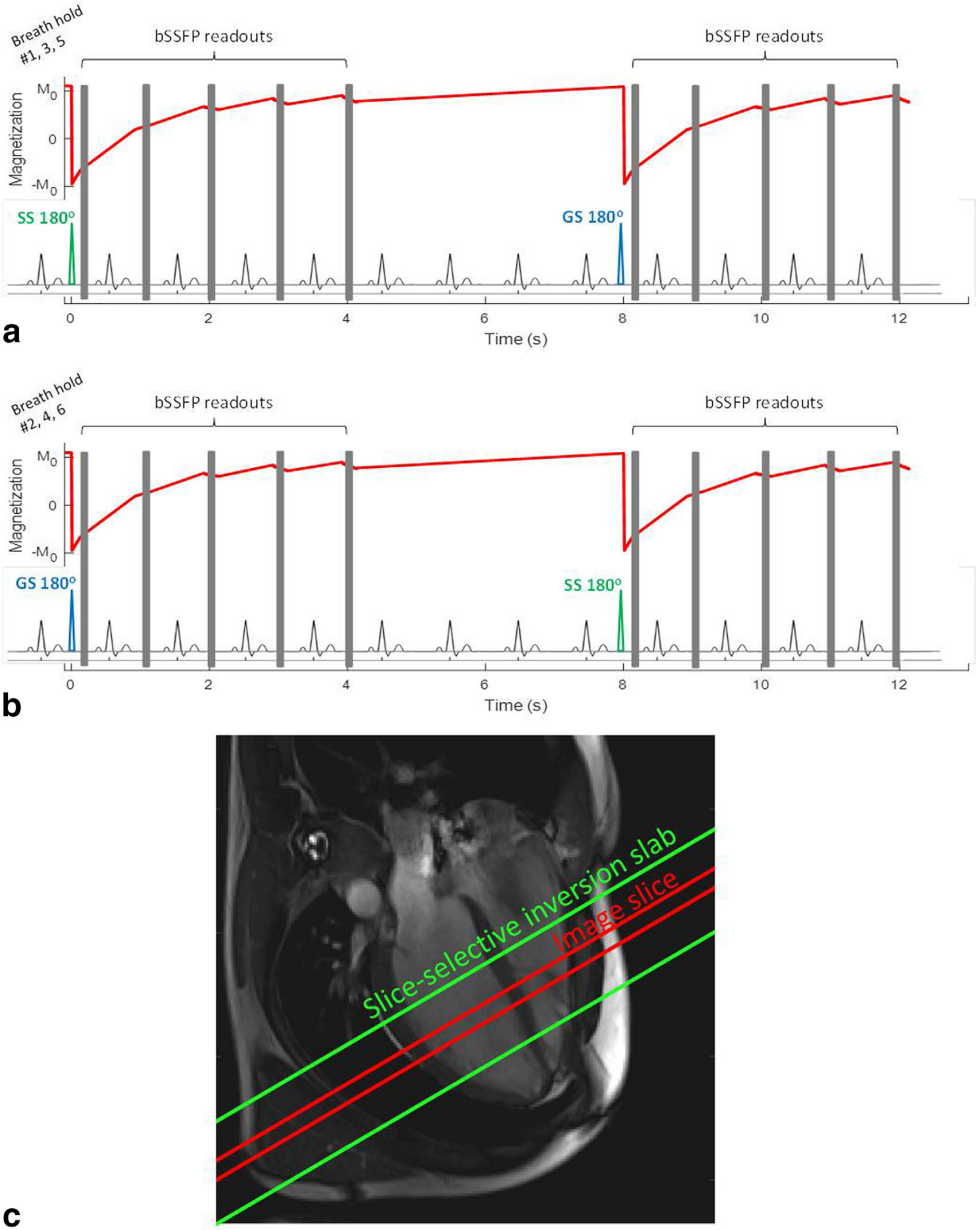
A schematic of the LL‐FAIR‐ASL pulse sequence over 13 heartbeats for breath‐holds 1, 3, and 5 (**a**) and breath‐holds 2, 4, and 6 (**b**). 180° pulses (one SS, one GS, within a single breath‐hold, the order of which is varied) are each followed by 5 bSSFP readouts (shown as gray boxes), separated by an R–R interval. The evolution of M_z_ throughout the sequence is shown in red. (**c**) A four‐chamber view of the heart with the position of the imaging slice in red, and the position of the SS inversion slab in green.

The LL‐FAIR‐ASL sequence was implemented on a 3 tesla (3 T) whole‐body scanner (TIM Trio, Siemens, Germany). The sequence consisted of two HS8 adiabatic inversion pulses 18, one SS, one GS with a duration of 10 ms, time/bandwidth product R = 40 and β = 3.45. Each inversion pulse was followed by a block of five cardiac triggered balanced steady‐state free precession (bSSFP) readouts 19. The readout consisted of LISA excitation pulses, which are Gaussian‐like, and included five initial ramp‐up pulses and a final half‐alpha “restore pulse.” The two inversion blocks were separated by a gap of three heartbeats to allow for some recovery of the magnetization, within a manageable breath‐hold, as shown in Figure 1, thus forming a MOLLI 5(3)5 regime. Both SS and GS inversions were collected in a single thirteen heartbeat breath‐hold. The ordering scheme of the sequence within this thirteen heart beat breath‐hold (SS‐GS or GS‐SS) was varied. Sequence parameters were based closely on those used successfully in MOLLI implementations and included a 35 ° excitation flip angle, image slice thickness of 8 mm, an initial TI of 115 ms (with the following values being 115 ms + RR, 115 ms + 2RR,…), TR/TE of 3 ms/1.5 ms, 320 mm field of view with 75% phase resolution and 6/8 partial Fourier, a 192 × 144 image matrix (interpolated to 384 × 288) and an acceleration factor of GRAPPA 2 20. All inversion pulses and readouts occurred during the diastolic phase of the cardiac cycle. This timing guaranteed that the signal recovery was not corrupted by through‐plane bulk cardiac motion, by restricting inversions and readouts to the most stable cardiac phase. Due to the inversion recovery nature of the sequence at least 15 s was allowed between scans to allow for full relaxation of the magnetization.

### Simulation

Bloch simulations of the sequence were performed in MATLAB (The Mathworks Inc., Natick, MA) with the acquisition parameters defined above and not accounting for magnetization‐transfer effects. The inversion pulses were simulated fully as HS8 pulses with the parameters matching those described above and the power set as per the scanner maximum output voltage to simulate the actual B_1_ power used on the scanner to ensure the adiabatic condition is met. The evolution of the longitudinal magnetization, M_z_, after inversion, and the influence of the readout blocks, based on these simulations, is shown in Figure 1. Simulations were performed for a range of *T*_1_s (100–2000 ms) and heart rates (40–140 bpm) to test the robustness of the sequence against variations in these parameters. To determine an appropriate value for the thickness of the SS inversion pulse, a range of thicknesses (8–35 mm) were simulated for the stated range of *T*_1_s, with a fixed image slice profile with thickness of 8 mm full width half maximum to investigate the interaction between the HS8 inversion pulse profile and the image slice profile. The inflow was not simulated, such that the performance of the sequence in measuring only the relaxation under the two different inversion conditions could be investigated to ensure that any difference measured in vivo was due to blood flow and not systematic error.

### Phantom Studies

A phantom imaging study was carried out to further investigate the performance of the LL‐Fair‐ASL sequence in the absence of flow. The data gathered were used to investigate the dependence of *T*_1_ and $T_{1}^{*}$ on heart rate with the LL‐FAIR‐ASL sequence by plotting these values against a range of simulated heart rates. The length of the gap between the two inversion blocks (measured in heartbeats) was also varied to investigate the effect of imperfect recovery before the second inversion. These experiments were carried out on an integrated calibration phantom distributed by Dr. S. Piechnik, Oxford, for the HCMR study (Clinicaltrials.gov ID: NCT01915615). The phantom was designed to monitor stability of *T*_1_ mapping techniques, and provides a *T*_1_ range of ∼300–3000 ms, and T_2_ ∼60–3000 ms. The phantom consists of nine test objects made of agar, carrageenan water gels doped with nickel chloride. The study was carried out on the 3 T scanner using a 32‐channel body receive array. The simulated ECG trigger was varied from 40 bpm to 140 bpm in 20‐bpm intervals. The resulting images were loaded into MATLAB for analysis, where regions of interest (ROIs) were drawn by hand within the confines of each tube and propagated throughout an image series to calculate the *T*_1_s of the samples.

### In Vivo Studies

ASL image series were acquired on 11 healthy volunteers (31 ± 7 years old; 71 ± 9 kg; two female) at 3T, in basal, mid‐ventricular, and apical short axis slices using the 32‐channel body receive array. All volunteers were recruited in accordance with the ethical practices of our institution and their informed consent obtained. Each of the volunteers was scanned twice, on separate days, making 22 scan sessions in total. The data from one subject was discarded due to excessive motion within the image series. In 17 of the 20 successful scans, the ASL acquisitions in the mid‐ventricular slice were repeated. B_0_ shimming was performed in a volume over the left ventricle (LV) covering all three slices. In each slice, the sequence as described in Figure 1 was run three times with the SS inversion block preceding the GS inversion block (called the SS‐GS ordering scheme), then run a further three times with the GS inversion block preceding the SS (GS‐SS), making for a total of six measurements, from which a single value of MBF could be calculated. An SS inversion thickness of 24 mm was used based on the results of the simulation and phantom scans described. This inversion slab and the imaging slice were positioned as described in Figure 1c. In all cases, ECG triggering and breath‐holding were used. No motion correction or image registration was used.

### Image Analysis

All image series were loaded into MATLAB and regions of interest (ROIs) were drawn by hand for both the left ventricular myocardium and the blood pool. For the myocardium, the epicardial and endocardial borders were drawn and the myocardium divided into segments as per the American Heart Association (AHA) model 21. The apparent *T*_1_ values ( $T_{1}^{*}$) for the myocardium for both the SS and global inversion blocks were calculated, as was the *T*_1_ of blood from the global data by using a three‐parameter least squares fit as described by:

(1) S(TI)=A−Be(−TIT1*)

where S was the signal intensity recorded at time TI and A, B, and $T_{1}^{*}$ were the fitted parameters. Where *T*_1_ values are reported, the correction described by Deichmann and Haase for FLASH images 22 and described in Equation [2] was used, although only strictly applicable in the small tip angle regime.

(2) $$T_{1}=T_{1}^{*}\left(\frac{B}{A}-1\right)$$

The phase data of the most fully recovered image from each inversion block were used to correct the polarity of the magnitude images on a pixel‐by‐pixel basis before fitting 23. The myocardial blood flow (MBF) for each slice was calculated from these data by the Belle quantification model 24:

(3) $$MBF=\;\frac{\lambda }{T_{1}^{blood}}\left(\frac{T_{1}^{GS}}{T_{1}^{SS}}-1\right)$$

where λ = 0.92 mL/g 25 is the blood‐tissue partition coefficient of water, $T_{1}^{\text{blood}}$ is the relaxation time of the blood pool and $T_{1}^{\text{GS}}$ and $T_{1}^{\text{SS}}$ are the values for the longitudinal relaxation time calculated for the myocardium, from the GS and SS experiment, respectively. For the in vivo study, the $T_{1}^{\text{GS}}$ and $T_{1}^{\text{SS}}$ values used were the observed $T_{1}^{*}$s. As has been discussed previously 26, 27, where the Deichmann‐Haase correction is used the ratio of the relaxation times remains constant $T_{1}{}^{\text{GS}}{/T}_{1}{}^{\text{SS}}=T_{1}^{*\text{GS}}/T_{1}^{*\text{SS}}$ as the fitted values of A and B should remain the same for both the GS and SS cases. Thus, use of the correction would have no effect on the final value of MBF. The use of $T_{1}^{*}$ is discussed further later in this work

### Reproducibility and Variability Analysis

The in vivo data were used to perform Bland‐Altman analysis 28 to assess reproducibility and variation. The mean difference in each case and the value of ± 1.96 times the standard deviations (SD) were calculated, which represent the upper and lower 95% confidence bounds. When normalized to the mean value of MBF, these values give the coefficient of repeatability for both the between‐session (CR_BS_) and within‐session cases (CR_WS_). The variability of the MBF estimates was assessed by the coefficient of variation (CV, equal to the ratio of the standard deviation to the mean) for the whole sample (CV_all_), and each subject between‐session (CV_BS_) and within‐session (CV_WS_).

### Segmental Analysis

Values of MBF were calculated for each of the 16 standard myocardial segments as defined but the AHA 21 to assess the viability of the ASL technique at segmental level. For each segment, these are reported as the mean and standard deviation across all the volunteers. The coefficient of variation (CV_seg_) is also reported for all segments.

## RESULTS

### Simulation

**Figure 2:**
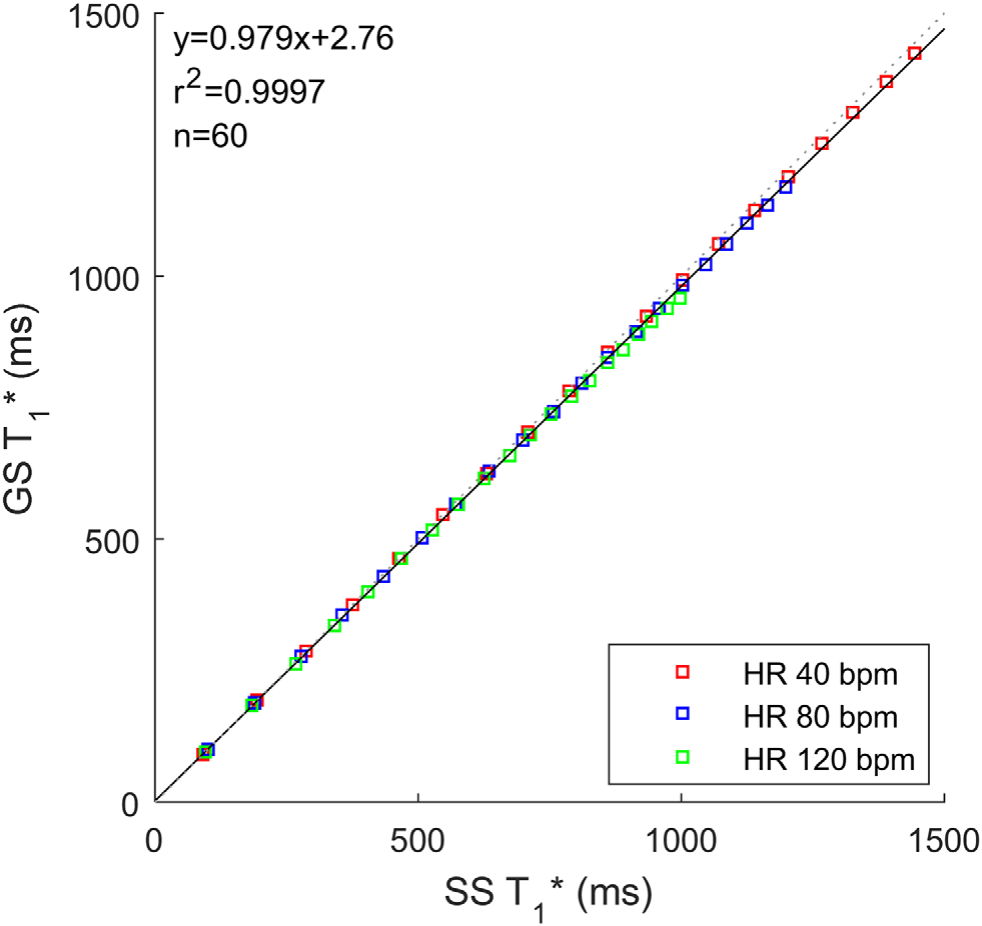
Correlation plot of the simulated SS and GS $T_{1}^{*}$ s, in the absence of flow, for heart rates of 40, 80, and 120 bpm and input *T*_1_ values from 100 to 2000 ms (100 ms steps).

$T_{1}^{*}$ data were plotted for heart rates of 40, 80, and 120 bpm and for the full range of input *T*_1_s. Figure 2 shows the simulated SS and GS $T_{1}^{*}$s to agree well in the absence of flow, with an R^2^ value of 0.9996 and both the $T_{1}^{*}$s shortening with increasing heart rate.

**Figure 3:**
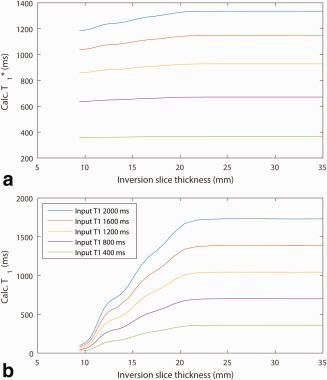
For an image slice thickness of 8 mm, the SS HS8 inversion pulse width, varied from 8 to 35 mm, is plotted against the simulated observed $T_{1}^{*}$ (**a**), and *T*_1_ (**b**) (following the Deichmann‐Haase correction) for input *T*_1_ values of 400 to 2000 ms (400 ms steps).

Figure 3 shows the results of simulations to determine an appropriate value for the thickness of the SS inversion pulse. Results for both *T*_1_ and $T_{1}^{*}$ are plotted showing the dependence of the calculation on the extent of the inversion. The values are stable down to an inversion thickness of 23 mm then decrease with decreasing thickness. The effect is more marked with *T*_1_ than $T_{1}^{*}$. For the input *T*_1_ of 1200 ms, approximately in the myocardial range, the $T_{1}^{*}$ drops from a maximum of 928 ms with the thickest inversion slice to 860 ms with the thinnest, a drop of 7%. The calculated *T*_1_, however, drops from 1043 ms with the thickest slice to just 63 ms with the thinnest, a drop of 94%.

### Phantom Studies

**Figure 4:**
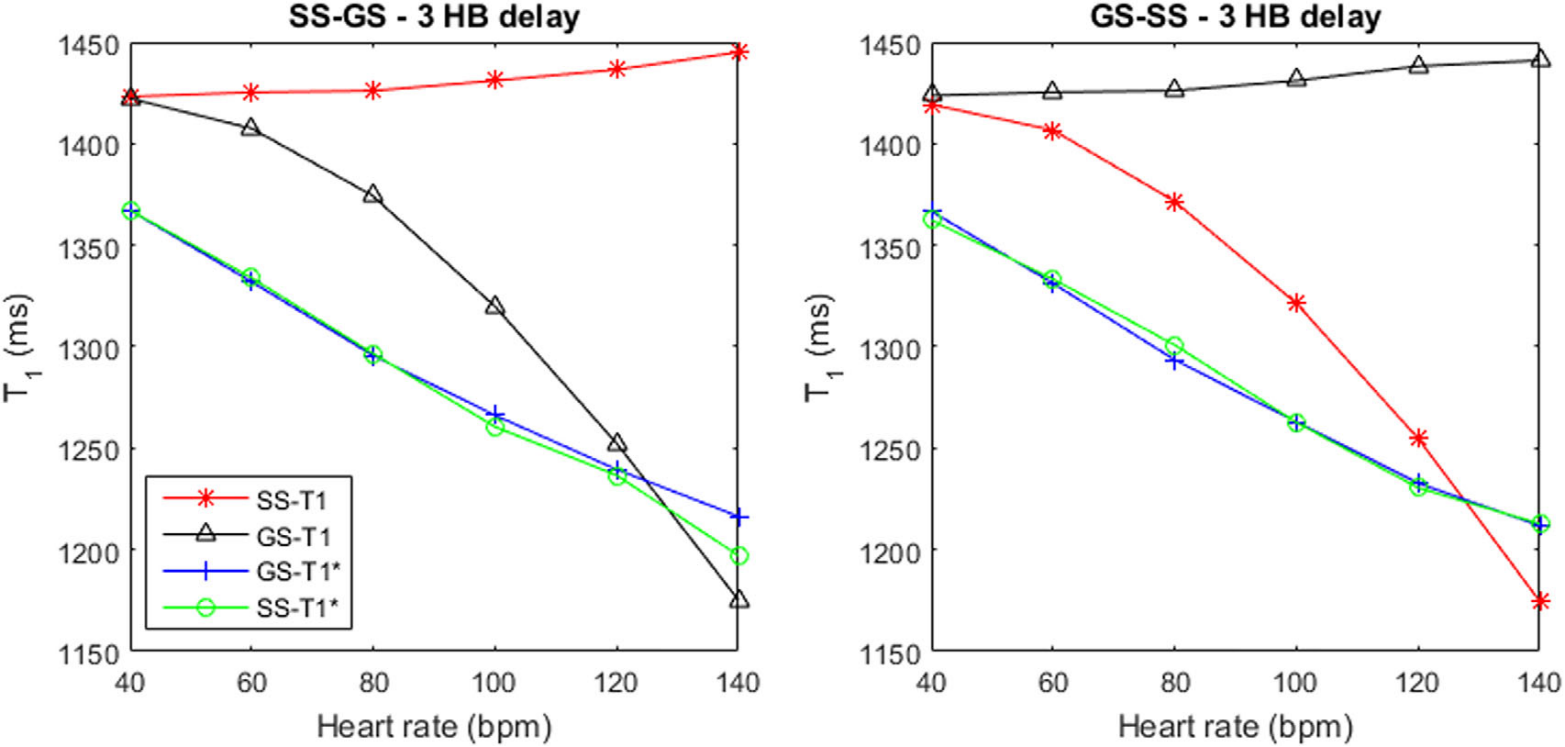
Measured $T_{1}^{*}$ and *T*_1_ of a cardiac phantom, for both the SS and GS inversion blocks plotted against simulated heart rate, from 40–140 bpm. There was a three heartbeat delay between the end of one block and the start of the next. The data are plotted for the SS‐GS (**a**) and the GS‐SS (**b**) ordering scheme.

An example plot of *T*_1_ and $T_{1}^{*}$ against simulated heart rate is shown in Figure 4 for a gap of three heart beats. The dependence of $T_{1}^{*}$ on heart rate can be clearly seen for both the SS‐GS and GS‐SS ordering schemes, decreasing steadily as the heart rate increases. The calculated *T*_1_, however, is stable for the first inversion block in the ordering scheme, whether SS or GS, with a value of 1431 ± 8 ms for the range of heart rates, but decreases rapidly with increasing heart rate, and, therefore, decreasing recovery time, for the second inversion block. Thus, the calculation of *T*_1_ is dependent on the ordering scheme, whereas the calculation of the $T_{1}^{*}$ s is not.

### In Vivo Studies

**Figure 5:**
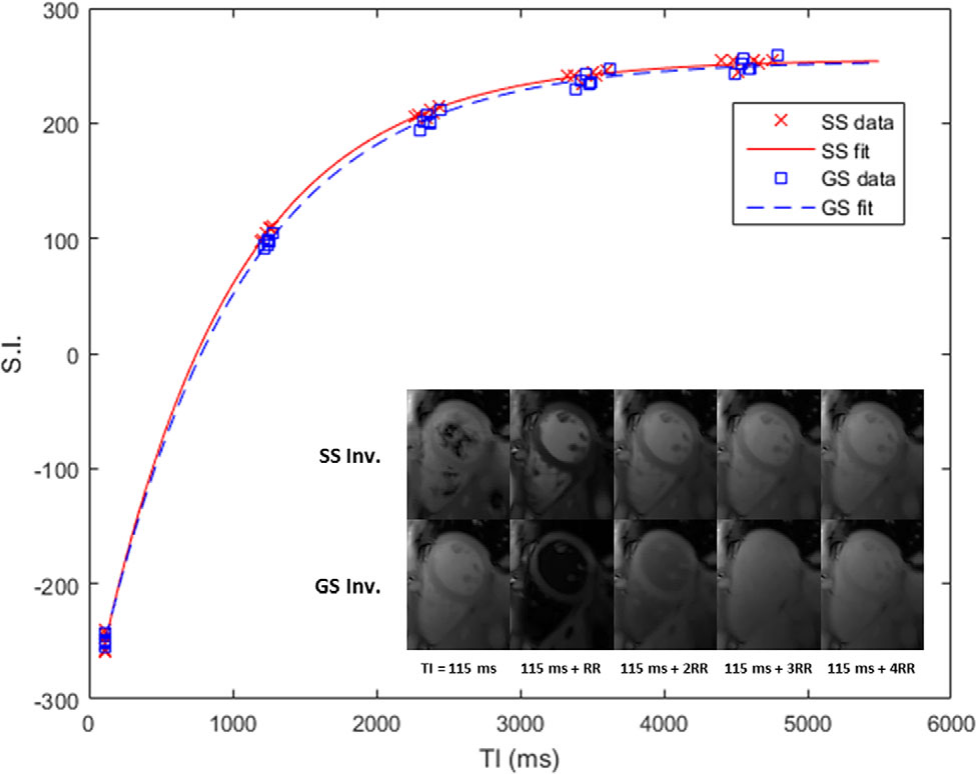
A typical pair of SS and GS inversion recovery curves with data points from six breath‐holds averaged over an ROI encompassing the left ventricular myocardium, and corresponding image series inset from one of the six breath‐holds.

**Figure 6:**
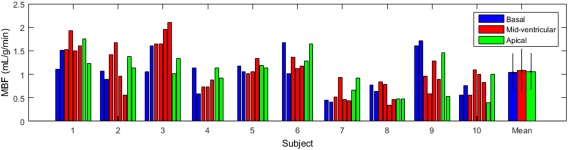
Calculated MBF results for both scan sessions for all 10 volunteers in basal, mid‐ventricular, and apical slices represented by blue, red, and green bars, respectively. Error bars denote the standard deviation.

Figure 5 shows a typical pair of *T*_1_ recovery curves, with inset SS and GS image series. The mean MBF across all the 10 volunteers and all 2 scan sessions was 1.04 ± 0.40 mL/g/min in the basal slice, 1.06 ± 0.46 mL/g/min in the mid‐ventricle and 1.06 ± 0.38 mL/g/min in the apical slice. The calculated values of MBF for each subject in each session, as well as the mean values, are presented in Figure 6. The data are presented for all scans in all three slices.

### Reproducibility and Variability Analysis

Values for the coefficients of variation and reproducibility, for both the between session and within session cases, are presented in Table 1. The CV across all scans was calculated as 39, 43, and 36% in the basal, mid‐ventricular, and apical slices, respectively. The CV_BS_ was 14, 16, and 17% in basal, mid, and apex; and the CV_WS_ was 13% in the mid‐ventricle. The CR_WS_ was 53% in the mid‐ventricle, compared with CR_BS_ values of 72, 61, and 85% in the base, mid, and apex.

**Table 1:** MBF Results from In Vivo Scans in Three Slices, Including the Mean Myocardial Blood Flow (MBF), the Standard Deviation and the CV Across all Scans and the CV and CR for Between Session (BS) and Within Session (WS) Cases

Slice	Mean MBF (mL/g/min)	SD (mL/g/min)	CV_all_ (%)	CV_BS_ (%)	CV_WS_ (%)	CR_BS_ (%)	CR_WS_ (%)
Basal	1.04	0.40	39	14		72	
Mid	1.06	0.46	43	16	13	61	53
Apical	1.06	0.38	36	17		85	

### Segmental Analysis

**Figure 7:**
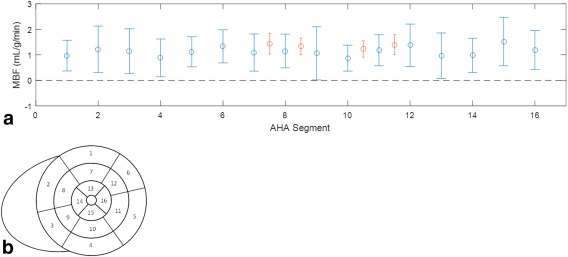
The mean ± standard deviation of MBF across all subjects for each myocardial segment as defined by the AHA (**a**), as described in (**b**), where segments 1–6 are the basal slice, 7–12 are the mid‐ventricular slice, and 13–16 are the apical slice. The data points in red are literature values in the septum and anterior, lateral and inferior walls of the mid–ventricle from Chareonthaitawee et al 2001 29.

The mean values and standard deviations of segmental MBF are reported in Table 2 along with the coefficient of variation, for all 16 segments. The range of mean segmental MBF values is 0.87–1.52 mL/g/min. These values are also plotted in Figure 7a, along with previous literature values of MBF in four segments in the mid‐ventricle (septum and anterior, lateral, and inferior walls) using ^15^O PET 29. The numbering of the segments is described in Figure 7b.

**Table 2:** Mean MBF Values for Each Segment, Where Segments 1‐6 Are the Basal Slice, 7‐12 Are the Mid‐ventricular Slice, and 13‐16 Are the Apical Slice

Segment	Mean MBF (mL/g/min)	SD (mL/g/min)	CV_seg_ (%)
1	0.97	0.6	62
2	1.22	0.91	75
3	1.14	0.88	77
4	0.88	0.74	84
5	1.12	0.59	53
6	1.33	0.65	49
7	1.09	0.73	67
8	1.15	0.66	57
9	1.06	1.04	98
10	0.87	0.51	59
11	1.18	0.61	52
12	1.38	0.83	60
13	0.97	0.89	92
14	0.98	0.67	68
15	1.52	0.95	63
16	1.19	0.76	64

## DISCUSSION

In this study, a noninvasive method for quantitatively measuring myocardial blood flow using arterial spin labelling was developed and tested by means of simulation, phantom experiment and in vivo studies. The variation and reproducibility of the method were then investigated in three short axis slices, where previous cardiac ASL studies have been restricted to the mid‐ventricle.

The Bloch simulations of the LL‐FAIR‐ASL sequence show good agreement between the SS and GS $T_{1}^{*}$s as presented in Figure 2. Thus, there is no dependence in the MBF calculation on error in the calculation of the $T_{1}^{*}$ ratio. This is true of the three simulated heart rates (40, 80, and 120 bpm), despite the expected shortened $T_{1}^{*}$ with increasing heart rate due to saturation from multiple high flip angle readouts, as seen in the simulated evolution of magnetization in Figure 1.

As observed in Figure 3, the calculation of $T_{1}^{*}$ and *T*_1_ is compromised at lower values of the inversion slice thickness relative to the imaging slice thickness. This occurs due to differences between the inversion profile and the image slice profile. The effect is far more marked in the calculation of *T*_1_ due to the reliance of the Deichmann‐Haase correction on inversion efficiency. At lower inversion thicknesses the efficiency is low due to the slice containing a mixture of inverted and noninverted spins. By comparison, $T_{1}^{*}$ has little dependence on inversion efficiency, providing further motivation for its use over *T*_1_. These data were used in the choice of inversion thickness for in vivo application of 24 mm.

Varying the simulated heart rate in the phantom scans demonstrates that the values of $T_{1}^{*}$ are far more stable than those of *T*_1_. Although $T_{1}^{*}$ is dependent on heart rate, as demonstrated in Figure 4, the $T_{1}^{*}$ values calculated for the SS and GS cases exhibit the same dependence, meaning heart rate does not bias the calculation of MBF as it is calculated from a ratio of the GS and SS $T_{1}^{*}$s. As discussed above, the *T*_1_ correction is highly dependent on the inversion efficiency, and in each ASL ordering scheme the second of the two inversions suffers from apparent poor efficiency due to insufficient time for relaxation between blocks, which is necessary for achievable breath‐holds. $T_{1}^{*}$ was used for all MBF calculations as the Deichmann‐Haase correction is only strictly applicable in a small angle regime and for inversion pulses applied to spins at equilibrium magnetization 22. $T_{1}^{*}$ is independent of inversion efficiency and, as stated earlier the ratio of GS to SS $T_{1}^{*}$s is the same as the ratio of GS to SS *T*_1_s.

The LL‐FAIR‐ASL method is attractive as it provides a noninvasive alternative to SPECT, PET, and first‐pass perfusion CMR. Using this method, MBF can be measured quantitatively in a single slice in six 13‐heartbeat breath‐holds. Each breath‐hold contains both SS and GS acquisitions, which ensures that the position of the heart and heart rate are the same for each pair. The ability to acquire an SS/GS pair in a single breath‐hold is an important feature of the LL‐FAIR‐ASL sequence, as the measured changes in $T_{1}^{*}$ are small and could easily be lost among systematic errors. The mean MBF, in the mid‐ventricular slice, of 1.06 ± 0.46 mL/g/min compares well with literature values, as presented in Table 3 29, 30, 31, 32, 33, 34, 35, 36.

**Table 3:** Comparison of LL‐FAIR Mid‐ventricular Resting MBF against Literature Values for Previous Cardiac ASL Methods, First‐Pass Perfusion CMR, and PET

Source	Method	Age (y)	Gender	Population	MBF_rest_ (mL/g/min)
LL‐FAIR‐ASL	ASL	31 ± 7	8 M/2 F	Healthy	1.06 ± 0.46
Zun 2009^36^	ASL		8 M/2 F	Healthy	1.36 ± 0.40
Zun 2011^35^	ASL	64 ± 11	10 M/19 F	CAD patients	0.97 ± 0.64
Wang 2010^34^	ASL	39 ± 9	10 M/1 F	Healthy	1.00 ± 0.55
Northrup 2008^33^	ASL	22 ± 2	5 M/3 F	Healthy	1.06 ± 0.19
Jerosch‐Herold 2008^31^	CMR ‐ First Pass	63 ± 10	11 M/19 F	Atherosclerosis	1.01 ± 0.22/0.91 ± 0.18^a^
Hsu 2006^30^	CMR ‐ First Pass	33 ± 4	2 M/8 F	Healthy	1.02 ± 0.22
Manabe 2009^32^	^82^Rb PET	29 ± 9	8 M/7 F	Healthy	0.99 ± 0.29/1.00 ± 0.25*
Chareonthaitawee 2001^29^	^15^O PET	46 ± 12	131 M/38 F	Healthy	1.33 ± 0.32

a Results from two separate exams on the same group of patients/volunteers.

While reproducibility and variability of similar techniques have been investigated in mice 37, 38, to our knowledge this has not been carried out in human myocardium. Resting MBF values for healthy volunteers have previously been shown to be heterogeneous in studies using PET 29 and first‐pass perfusion CMR 39. The high observed values for CV_all_ across all subjects, 39, 43, and 36% for basal, mid, and apical slices, respectively, show that our results reflect this heterogeneous nature. This variability, calculated as the coefficient of variation is comparable to similar measures reported for preclinical cardiac ASL 37, 38.

The values of CV_BS_ and CV_WS_ calculated for individual subjects were all below the values reported for CV_all_ except for a single apical slice which gave a CV_BS_ of 43%. However, the mean values of CV_BS_ for each slice and CV_WS_ for the mid slice only, are significantly lower than CV_all_, with a maximum of 17%. This shows that the variation in results exhibited by an individual subject in each case is much less than across the population as a whole. The CV_BS_ shows the variation due to the method, plus re‐setup effects such as repositioning, re‐localization, re‐shimming etc. The CV_WS_ primarily reflects the methodological effects.

The Bland‐Altman analysis showed the mean difference in both the between‐session case for all three slices and the within‐session case, for the mid slice only, to be close to zero and all bar one of the data points to lie within the ± 1.96 SD bounds. The values of CR give an indication of the change in MBF required to be detected above systematic errors. The between‐session CRs of 72, 61, and 85% for the basal, mid, and apical slices, show the level of repeatability expected across repeat scans. They show the change in MBF required to show a difference over time and is useful to consider if planning a longitudinal study in a patient group. The within‐session CR of 53% for the mid‐ventricular slice gives a useful indication of the detectable change in MBF. Thus, a change in MBF would be reliably detectable when rising from the mean mid‐ventricular MBF, measured with LL‐FAIR‐ASL, of 1.08 mL/g/min to a value of 1.74 mL/g/min between‐session and 1.65 mL/g/min within‐session. During vasodilator stress, the change in MBF in nonischemic segments of the heart has been shown to be between 300 and 420% 35, 40, 41, or between 3.24 and 4.54 mL/g/min based on the mean mid‐ventricular MBF reported here. Therefore, the expected change in global MBF under stress should be detectable with the LL‐FAIR‐ASL method.

Segmental MBF is an important measure in disease, as it allows investigation of MBF changes in the arterial territories of the myocardium. Previous publications using PET 29 and ASL 35 have commented on the spatial heterogeneity of resting MBF within a slice. The relative dispersion, here called the coefficient of variation, was reported as 13% with PET and 68% (range, 11% to 152%) with ASL, compared with 67% (with a range of 49% to 98%) with LL‐FAIR‐ASL. This difference in error between the ASL methods and PET is also reflected in the data plotted in Figure 7 and is perhaps expected given the inherently low signal‐to‐noise ratio of the method and the low resolution of the PET method, noted as a limitation by the authors and reported as 8.43 × 8.33 × 6.6 mm^3^ full‐width half maximum compared with 1.7 × 2.2 × 8 mm^3^ with LL‐FAIR‐ASL. However, given the expected increase in MBF under stress, the difference in perfusion reserve between ischemic and normal segments, should be detectable.

This study served to validate the LL‐FAIR‐ASL technique for measurement of resting MBF in healthy humans. Future studies will be required to validate the method in patient populations under stress and compare the results with standard methods such as first‐pass perfusion CMR. Image registration methods may be used to compensate for residual motion within breath‐holds, particularly in studies involving patients. In addition, more complex models for quantification of MBF, taking account of such effects as arterial transit time, the partial inversion of the blood pool during SS inversion and the perturbation of the inversion recovery by the readouts could be implemented. However, these studies are beyond the scope of this work.

## CONCLUSIONS

The LL‐FAIR‐ASL sequence for quantitative measurement of MBF was shown to be robust and efficient when performed in vivo. The method is completely noninvasive, not requiring contrast agent, and provides resting MBF values in healthy volunteers which compare well with the literature and display the previously reported heterogeneity within healthy volunteer groups. MBF values are reported globally in three slices with a cardiac ASL technique for the first time. The variability of the method was shown to compare favorably with published values in similar techniques used in preclinical imaging but, to our knowledge, has not previously been performed in human volunteers. The method was shown to be sensitive enough to detect MBF increase under stress conditions in future studies. The presented results should prove useful in the planning of clinical research studies using this sequence to quantitatively measure MBF at rest and under stress in healthy volunteers or in patient groups.
